# Risk for Recurrence After Liver Resection in Patients with Hepatitis C Virus-Related Hepatocellular Carcinoma Detected After Sustained Virological Response by Direct-Acting Antivirals: A Retrospective Multicenter Study

**DOI:** 10.3390/cancers17121946

**Published:** 2025-06-11

**Authors:** Shogo Tanaka, Takehiro Noda, Koji Komeda, Satoshi Yasuda, Masaki Ueno, Haruki Mori, Hisashi Kosaka, Ryo Morimura, Hiroji Shinkawa, Naoko Sekiguchi, Hisashi Ikoma, Takeaki Ishizawa, Masaki Kaibori

**Affiliations:** 1 Department of Hepato-Biliary-Pancreatic Surgery, Graduate School of Medicine, Osaka Metropolitan University, Osaka 545-8585, Osaka, Japan; 2 Department of Gastroenterological Surgery, Graduate School of Medicine, Osaka University, Suita 565-0871, Osaka, Japan; 3 Department of General and Gastroenterological Surgery, Osaka Medical and Pharmaceutical University, Takatsuki 569-8686, Osaka, Japan; 4 Department of Surgery, Nara Medical University, Kashihara 634-8521, Nara, Japan; hi22zd@naramed-u.ac.jp; 5 Second Department of Surgery, Wakayama Medical University, Wakayama 641-8509, Wakayama, Japan; 6 Department of Surgery, Shiga University of Medical Science, Otsu 520-2192, Shiga, Japan; 7 Department of Hepatobiliary Surgery, Kansai Medical University, Hirakata 573-1010, Osaka, Japankaibori@hirakata.kmu.ac.jp (M.K.); 8 Department of Surgery, Kyoto Prefectural University of Medicine, Kyoto 602-8566, Kyoto Prefecture, Japan; morimura@koto.kpu-m.ac.jp (R.M.);

**Keywords:** antiviral agents, hepatocellular carcinoma, hepatitis C virus

## Abstract

**Backgrounds**: Direct-acting antiviral (DAA) therapy, which achieves a high sustained virological response (SVR) rate, has been established as a standard treatment for patients with hepatitis C virus (HCV) infection. However, the risk factors for postoperative recurrence in patients with HCV-related hepatocellular carcinoma (HCC) detected after the achievement of an SVR by DAAs are unknown. **Methods**: The clinical records of 95 patients with initial HCV-related HCC detected after DAA-SVR achievement, who underwent liver resection between September 2014 and December 2020, were retrospectively reviewed. Patients with major vascular invasion and/or SVR achievement induced by interferon-based therapy were excluded. In this study, the patients were divided into two groups according to their alcohol intake status: without alcohol abuse (<80 g of ethanol each day for at least 5 years, n = 85) and with (continuous) alcohol abuse (n = 10). The risk factors for recurrence after liver resection were investigated, with special reference to the alcohol intake status. **Results**: The 3- and 5-year disease-free survival (DFS) rates after liver resection were 68.7% and 55.3%, respectively. Univariate and multivariate analyses identified alcohol abuse [hazard ratio (HR) 3.36, *p* = 0.004] and tumor size (HR 2.53, *p* = 0.010) as independent risk factors for postoperative recurrence. The 3- and 5-year postoperative DFS rates were 72.2% and 61.5% for patients without alcohol abuse and 40.0% and 13.3% for those with alcohol abuse (*p* = 0.001). **Conclusions**: Continuous alcohol abuse is a risk factor for recurrence after surgery of HCC detected after the achievement of DAA-SVR.

## Simple Summary

The risk factors for postoperative recurrence in patients with hepatitis C virus (HCV)-related hepatocellular carcinoma (HCC) detected after the achievement of a sustained virological response (SVR) by direct-acting antivirals (DAAs) are unknown. The clinical records of 95 patients with initial HCV-related HCC detected after DAA-SVR achievement, who underwent liver resection, were retrospectively reviewed. The 3- and 5-year disease-free survival (DFS) rates after liver resection were 68.7% and 55.3%, respectively. Univariate and multivariate analyses identified alcohol abuse and tumor size as independent risk factors for postoperative recurrence. Continuous alcohol abuse is a risk factor for recurrence after surgery of HCC detected after the achievement of DAA-SVR.

## 1. Introduction

Hepatocellular carcinoma (HCC) is the third leading cause of cancer-related death worldwide [1]. In Japan, viral hepatitis remains the leading cause of HCC; however, the decrease in the prevalence of hepatitis C virus (HCV)-related HCC has changed the distribution of the etiology [2]. HCV accounted for >70% of the total number of HCCs; however, in recent years, this percentage has decreased to 20–40% [3,4]. Moreover, the high recurrence rate after curative treatment for HCV-related HCC remains an issue, with a cumulative recurrence rate of 60–90% five years after hepatic resection [5,6]. Previous studies have indicated that a sustained virologic response (SVR) induced by interferon-based therapy (IBT) decreases HCC recurrence after liver resection [7].

Direct-acting antiviral (DAA) therapy, an alternative to IBT, which achieves a high SVR rate (≥95%), has been established as a standard treatment for patients with HCV infection [8,9,10]. DAA therapy has gained popularity since insurance coverage was established for the treatment of HCV infection on 2 September 2014, in Japan. Our previous report indicated that the disease-free survival (DFS) rate after liver resection for HCC is better in patients who achieved DAA-SVR than in those without treatment or with a non-SVR [11]. However, at present, nearly all patients with HCV-related hepatitis received DAA therapy, achieving an SVR, which was not obtained in patients who had severe comorbidities or refused treatment [12]. Therefore, investigating the risk for postoperative recurrence in patients who achieved DAA-SVR is essential.

In this study, we analyzed the risk factors for postoperative recurrence in patients with HCV-related HCC that was detected after achieving DAA-SVR.

## 2. Materials and Methods

### 2.1. Patients

**Figure 1:**
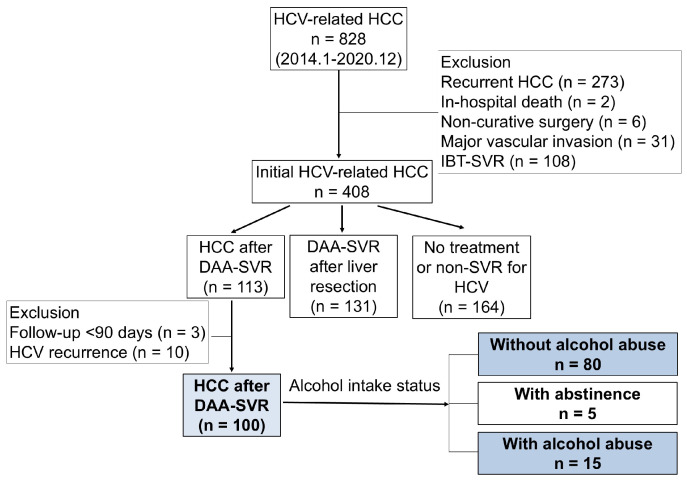
Schema of the study. DAA, direct-acting antiviral; HCC, hepatocellular carcinoma; HCV, hepatitis C virus; IBT, interferon-based therapy; SVR, sustained virological response. “HCC after DAA-SVR”: detection of hepatocellular carcinoma after the achievement of a sustained virological response by direct-acting antiviral therapy. “DAA-SVR after liver resection”: achievement of a sustained virological response by direct-acting antiviral therapy after curative resection.

Between September 2014 and December 2020, 828 patients with HCV-related HCC underwent liver resection at eight university hospitals. Among these patients, 424 were excluded from the study for undergoing surgery for recurrent HCC (n = 273), in-hospital death (n = 2), noncurative surgery (n = 6), major vascular invasion (infiltration to the first branch of the portal vein and/or major hepatic vein, n = 3), or IBT-induced SVR (n = 108). Thus, 408 patients underwent liver resection for the treatment of initial HCV-related HCC. Among them, 113 achieved a DAA-SVR before HCC detection. Following the exclusion of patients with HCV recurrence after the DAA-SVR (n = 10), follow-up <90 days after surgery (n = 3), and abstinence (n = 5), 95 patients were retrospectively reviewed (Figure 1). Of the 95 patients, 10 had a history of alcohol abuse. This retrospective study was conducted following the mandates of the Helsinki Declaration and ethical committee guidelines of our institution (Osaka Metropolitan University, registration no. 2023-051).

### 2.2. Criteria for Hepatic Resection

In general, the criteria for hepatic resection were in accordance with the criteria of Makuuchi et al. [i.e., based on the presence or absence of ascites, the serum total bilirubin level, and the indocyanine green retention rate at 15 min (ICGR15)] [13]. Ascites were either not detected or controllable with diuretics, and the serum total bilirubin concentration was <2.0 mg/dL. Patients with a serum total bilirubin concentration between 1.1 and 1.9 mg/mL or those with ICGR15 ≥ 30% were selected for limited resection or enucleation. In patients with a serum total bilirubin concentration of ≤1.0 mg/dL, two-thirds of noncancerous liver parenchyma could be removed if ICGR15 was ≤10%, and less than one-third could be resected if this value was 10–19%, whereas those with ICGR15 of 20–29% underwent Couinaud’s segmentectomy or less. The hepatic anatomy and type of hepatic resection were classified according to the Brisbane 2000 Terminology of Liver Anatomy and Resections [14]. The treatment plan was decided by the Cancer Board, and liver resection in general was the first choice for initial HCC with good liver function and three or fewer tumors, regardless of tumor size, in accordance with the Clinical Practice Guidelines for Hepatocellular Carcinoma (5th JSH-HCC guidelines) [15].

### 2.3. Interferon-Free DAA Therapy for Chronic HCV Treatment

The inclusion criteria for DAA therapy were as follows: presence of serum antibodies to HCV and serum HCV RNAs and absence of hepatitis B surface antigen (HBsAg) and severe comorbidities. Among the 95 patients in the DAA group, the planned DAA regimens were as follows: sofosbuvir + ledipasvir for 12 weeks (n = 45 [with ribavirin, n = 11; without ribavirin, n = 34]), asunaprevir + daclatasvir for 24 weeks (n = 30), ombitasvir + paritaprevir + ritonavir for 12 weeks (n = 9), elbasvir + grazoprevir (n = 6), and glecaprevir + pibrentasvir (n = 5). All 95 patients completed the DAA therapy without experiencing side effects. The SVR persisted for ≥24 weeks after the commencement of DAA therapy in all patients.

### 2.4. Definition

Patients who took >80 g of ethanol each day for at least 5 years were defined as having alcohol abuse [16]. For this study, the cohort was divided into three groups according to the alcohol intake status: patients without alcohol abuse, patients who were once alcohol abusers but were abstinent at the time of HCC diagnosis (patients with abstinence), and patients who were alcohol abusers at the time HCC diagnosis (patients with alcohol abuse). The tumor stage was classified according to the Union for International Cancer Control (UICC) [17]. The recurrence time was defined as the duration from the day of liver resection to the day when recurrence was diagnosed based on imaging assessments. The post-recurrence survival time was measured from the date of recurrence at radiology until the date of death or last follow-up (31 December 2022). The overall survival (OS) time was defined as the interval from liver resection to the date of death or last follow-up [5,18].

### 2.5. Data Collection

The clinical data of all patients were collected prospectively from electronic chart records and are summarized in Table 1.

### 2.6. Pathology

Intraoperatively, the liver tissue sample was cut serially into 5 mm thick tissue blocks, fixed in 10% formalin, and stained with hematoxylin and eosin. All specimens were histopathologically evaluated. For non-HCC liver tissue, the histological activity index (HAI) was used, with some modifications [19,20], to evaluate the degree of hepatic fibrosis (hepatic fibrosis score). This score was defined as follows: 1, portal fibrous expansion; 2, portal–portal septa without architectural distortion; 3, portal–central septa with architectural distortion; and 4, cirrhosis.

### 2.7. Follow-Up Evaluations

All patients attended follow-up appointments every 3 months after discharge for 5 years following hepatic resection and every 3–6 months thereafter [20]. Follow-up evaluations included physical examination, liver function tests, chest radiography or chest-computed tomography to check for pulmonary metastases; and ultrasonography, computed tomography, and magnetic resonance imaging to check for recurrence in the remnant liver or other organs. Bone metastases were diagnosed by magnetic resonance imaging and/or bone scintigraphy. Patients who had a recurrence were treated with appropriate therapeutic modalities such as curative treatment or transcatheter arterial chemoembolization. These treatments were selected based on the patient’s general condition, tumor characteristics, and liver function at the diagnosis of HCC recurrence.

### 2.8. Statistical Analyses

Continuous variables are expressed as median values (range) and were compared between groups using the Mann–Whitney *U* test. The differences between categorical variables were analyzed using the chi-squared test or Fisher’s exact test. The Kaplan–Meier method was used to calculate DFS and OS. Differences in the rates between the selected groups were evaluated using a log-rank test. To estimate the risk factors for recurrence after liver resection, Cox’s proportional hazard model with stepwise variable selection was employed. In this study, continuous values were converted into categorical data using their median values. Variables with a *p*-value < 0.05 in the univariate analysis (log-rank test) were entered into the multivariate analysis. A *p*-value < 0.05 was considered significant. All statistical analyses were performed using IBM SPSS Statistics version 26.0 (IBM Corp., Armonk, NY, USA).

## 3. Results

### 3.1. Clinicopathological Characteristics of Patients with HCV-Related HCC Detected After DAA-SVR

Among all populations, the median follow-up period was 1,370 (range, 146–2929) days. The median duration from the start of DAA-SVR to HCC diagnosis was 788 (range, 27–2269) days (Table 1). The study included 85 patients without alcohol abuse and 10 patients with alcohol abuse. In the liver function tests, one patient had been classified into Child–Pugh class B. Among the surgery-related factors, the proportion of patients who underwent minimal invasive surgery (laparoscopic or robot-assisted liver resection) was high (78% [74/95]). Among the tumor-related factors, the median tumor size was 2.0 cm, and 47% (n = 45), 39% (n = 37), and 14% (n = 13) of the patients had UICC stages IA, IB, and II, respectively. Pathologically, liver cirrhosis was present in 35 (37%) patients.

### 3.2. Postoperative Recurrence

**Figure 2:**
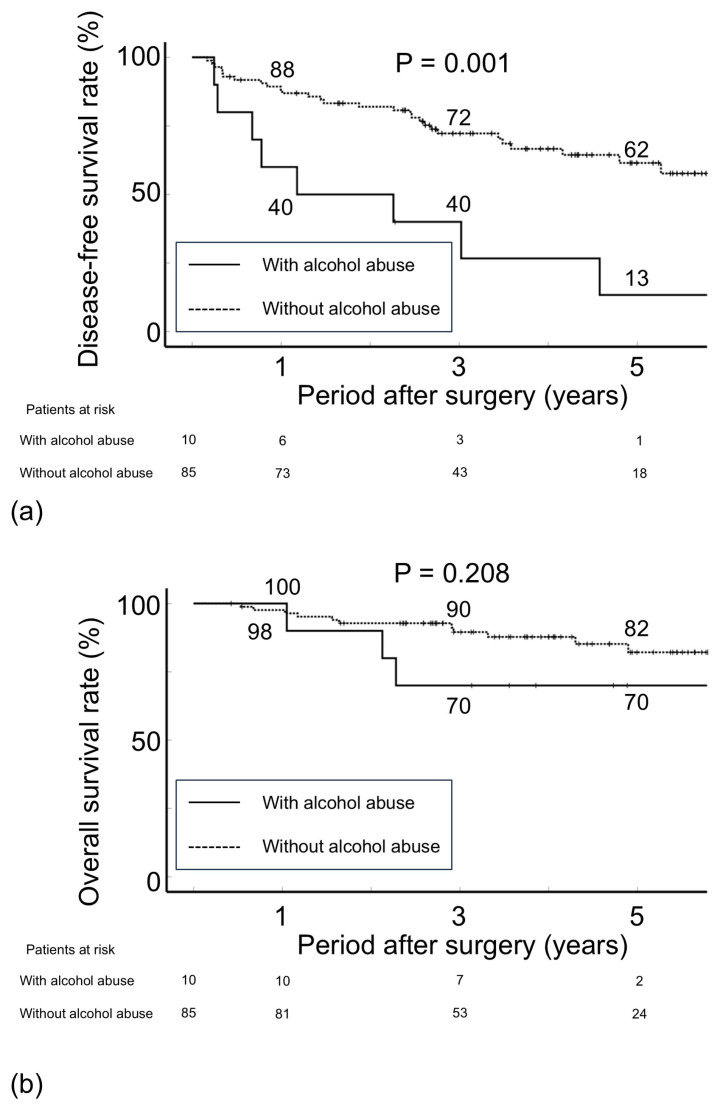
Disease-free survival rate (**a**) and overall survival rate (**b**) after liver resection for patients with hepatitis C virus-related hepatocellular carcinoma detected after the achievement of a sustained virological response according to alcohol intake status.

During the follow-up period, 36 (38%) patients had postoperative recurrence. The 3- and 5-year DFS rates after liver resection were 68.7% and 55.3%, respectively. Alcohol abuse, alanine aminotransferase (ALT, ≥18 IU/L), and tumor size (≥2 cm) were possible factors for postoperative recurrence (Table 2). In total, 8 of 10 patients with alcohol abuse and 28 of 85 patients without alcohol abuse had postoperative recurrence. The 3- and 5-year DFS rates after liver resection were 40.0% and 13.3% for patients with alcohol abuse and 72.2% and 61.5% for those without alcohol abuse (*p* = 0.001, Table 2 and Figure 2a). There was no significant difference in initial recurrence sites or treatment for recurrence (Table 3). In addition, 2 of 8 patients with alcohol abuse and 9 of 28 patients without alcohol abuse died after recurrence. There was no difference in post-recurrence survival rate among the two groups. The serum levels of aspartate aminotransferase (AST) and ALT were higher in patients with alcohol abuse (n = 10) than in those without alcohol abuse (n = 85, Table 4). However, no difference in tumor-related variables and pathological findings was noted between the two groups. During the follow-up period, in addition to the above 11 patients, 5 patients died of other malignancies or liver-unrelated diseases. The OS rate 3/5 years after surgery was 70.0%/70.0% for patients with alcohol abuse and 89.5%/82.1% for those without alcohol abuse (*p* = 0.208, Figure 2b).

## 4. Discussion

The dissemination of DAA therapy has drastically changed the treatment of patients with HCV infection. With DAA therapy, SVR was achieved in 95–97% of patients with compensated cirrhosis [8,9,10] and 85–90% of those with disease at more advanced stages [21,22]. With DAA-SVR, the 1-year HCC occurrence rate ranged from 0% to 2.2% [23,24,25,26]. The HCC occurrence rate was lower for patients who achieved DAA-SVR than for those without treatment for HCV infection or with non-SVR [24,27] and was not significantly different from the rate obtained with IBT-SVR [28,29]. As such, DAAs are now administered to patients with HCV in the absence of special circumstances [12]. Therefore, in the future, it will be important not to compare outcomes with and without DAA-SVR [11,12,30] and to study DAA-SVR cases in detail. In this study, an alcohol abuse status at the time of HCC diagnosis was found to be an independent risk factor for HCC recurrence in patients who achieved DAA-SVR.

Excess alcohol consumption is one of the major causes of HCC occurrence [31]. Previous studies have advocated that alcohol consumption had a synergistic effect on HCC development in patients with chronic viral hepatitis [31,32] and increased the HCC risk even in patients after IBT- and/or with DAA-SVR [33,34]. In this study, no difference in tumor-related variables or pathological examination data was found, and the serum transferase levels were higher in patients with alcohol abuse than in those without alcohol abuse. On the basis of this evidence, even after the achievement of DAA-SVR, continuous alcohol abuse might induce multicentric occurrence [35], rather than intrahepatic recurrence [36]. However, whether abstinence could reduce postoperative recurrence in patients with HCC detected after achieving DAA-SVR is controversial. Furthermore, there was no difference in post-recurrence survival between patients with and without alcohol abuse, which may be due to the lack of differences in Child–Pugh class and tumor-related variables, as described by Facciorusso, et al. [18].

In this study, a tumor size ≥2.0 cm was an independent risk factor for postoperative recurrence. As regards tumor-related variables, tumor size, microvascular invasion, and multiple tumors were risk factors for HCC recurrence after liver resection [37,38,39], all of which affect the tumor stage [17]. This study excluded patients with major vascular invasion. In addition, patients who received DAA therapy are generally followed routinely, leading to the detection of small HCCs (median size, 2.0 cm). Microvascular invasion and multiple tumors were not a risk for recurrence. Berardi et al. [40] reported that a tumor diameter >2 cm increased the recurrence risk. According to the UICC classification, HCCs ≤2 cm are considered “early HCC” and are classified as T1a tumors. Therefore, even after achieving DAA-SVR, a close follow-up is necessary to detect HCCs even with a small diameter, in addition to liver function tests and the analysis of tumor markers.

This study has several limitations. First, this study has a retrospective design. Second, the occurrence rate of HCC after DAA-SVR was low, and the coverage period from insurance in Japan is short; thus, the number of patients in the cohort was small. Third, no patients had stage III/IV disease, likely because the patients who received DAA therapy attended routine follow-up evaluations. Thus, the effects of DAA-SVR on advanced HCC after liver resection could not be assessed.

## 5. Conclusions

Alcohol abuse and tumor size appeared as independent risk factors for postoperative recurrence of HCV-related HCC after DAA-SVR. Liver function was worse in patients with alcohol abuse than in those without alcohol abuse or with abstinence. However, more studies are needed to investigate whether abstinence could reduce the postoperative recurrence rates.

## Figures and Tables

**Table 1:** Clinical characteristics of 95 patients with HCC after DAA-SVR.

Variables	Data		Variables		Data
Background			Surgery-related variables		
Age (y.o.)	72	(45–88)	Tumor location		
Sex (male/female)	56/39		Anterolateral segments	57	(60)
History of IBT	28	(30)	Posterosuperior segments	38	(40)
Duration between DAA–SVR and detection of HCC (days)	788	(27–2269)	Segmentectomy or more	31	(33)
BMI (Kg/m^2^)	23.3	(17.0–34.8)	Minimal invasive surgery	74	(78)
Status of alcohol intake			Operation time (min)	251	(86–641)
Without alcohol abuse	85	(89)	Bleeding (cc)	50	(0–2340)
With alcohol abuse	10	(11)			
Diabetes mellitus	24	(25)	Tumor-related variables		
			AFP (ng/mL)	6	(1.0–25,452)
Preoperative liver function tests			PIVKA-II (U/mL)	27	(1.0–15,600)
Total bilirubin (mg/dL)	0.7	(0.2–2.2)	Tumor size (cm)	2	(0.7–7.5)
Albumin (g/dL)	4.2	(3.3–5.0)	Multiplicity	12	(13)
Prothrombin activity (%)	91	(59–125)	Macrovascular invasion	2	(2)
ICGR 15 min (%)	13	(1.0–37.1)	UICC stage 1A/1B/2	45/37/13	
Child A/B	94/1				
Platelets (×10^4^/μL)	12.3	(4.1–34.9)	Histology		
AST (IU/L)	26	(12–56)	Poorly differentiated HCC	15	(16)
ALT (IU/L)	18	(7–66)	Microvascular invasion	15	(16)
			Liver cirrhosis	35	(37)

Note: data are presented as median (ranges) or number (percentages). AFP, α-fetoprotein; ALT, alanine aminotransferase; AST, aspartate aminotransferase; BMI, body mass index; DAA, direct-acting antiviral; HCC, hepatocellular carcinoma; IBT, interferon-based therapy; ICGR 15 min, indocyanine green retention rate at 15 min; PIVKA-II, protein induced by vitamin K absence or antagonist-II; SVR, sustained virological response; UICC, International Union Against Cancer.

**Table 2:** Risk factors for postoperative recurrence in patients with HCC detected after DAA-SVR.

			DFS			Multivariate Analysis		
Variables		n	3 Years	5 Years	*p* Value	HR	(95% CI)	*p* Value
Background								
Age					0.197			
	≥72 y.o.	49	69.1	55.7				
	<71 y.o.	46	68.3	44.9				
Sex (male/female)					0.080			
	Male	56	66.8	45.2				
	Female	39	71.9	71.9				
History of IBT					0.745			
	Presence	28	78.0	57.0				
	Absence	67	64.5	55.1				
Duration between DAA-SVR and detection of HCC <1 year					0.306			
	<1 year	24	64.2	49.4				
	≥1 year	71	70.0	56.7				
BMI					0.722			
	≥23.3 Kg/m^2^	48	70.7	47.1				
	<23.3 Kg/m^2^	47	66.6	60.3				
Status of alcohol intake					0.001	3.36	(1.49–7.61)	0.004
	Without alcohol abuse	85	72.2	61.5				
	Alcohol abuse	10	40.0	13.3				
Diabetes mellitus					0.321			
	Presence	24	57.6	48.0				
	Absence	71	71.9	57.8				
Preoperative liver function tests								
Total bilirubin					0.28			
	≥0.7 mg/dL	56	64.2	50.3				
	<0.7 mg	39	74.6	61.7				
Albumin					0.602			
	≤4.2 g/dL	52	68.9	58.9				
	>4.2 g/dL	43	68.5	49.7				
Prothrombin activity								
	≤91%	52	60.7	45.8				
	>91%	48	75.5	65.8				
ICGR 15 min					0.258			
	≥13.0%	52	66.8	49.9				
	<13.0%	43	71.2	60.5				
Platelets					0.312			
	≤12.3 × 10^4^/μL	48	65.5	49.4				
	>12.3 × 10^4^/μL	47	69.7	59.6				
AST					0.076			
	≥26 IU/L	48	66.1	44.5				
	<26 IU/L	47	71.1	71.1				
ALT					0.008	2.05	(0.94–4.48)	0.072
	≥18 IU/L	52	63.0	41.5				
	<18 IU/L	43	75.8	75.8				
Surgery-related variables								
Tumor location					0.982			
	Anterolateral segments	57	66.0	55.7				
	Posterosuperior segments	38	72.4	55.7				
Segmentectomy or more					0.438			
	Performed	31	76.5	61.5				
	Not performed	64	64.8	52.5				
Approach					0.617			
	Minimal invasive surgery	74	68.4	53.9				
	Open surgery	21	70.0	61.3				
Operation time					0.616			
	≥251 min	48	69.1	59.4				
	<251 min	47	68.3	51.2				
Bleeding					0.528			
	≥50 cc	56	66.9	50.7				
	<50 cc	39	70.7	59.4				
Tumor-related variables								
AFP					0.113			
	≥6 ng/mL	49	63.3	46.6				
	<6 ng/mL	46	74.7	66.5				
PIVKA-II					0.354			
	≥27 U/mL	50	61.7	48.2				
	<27 U/mL	45	71.7	58.5				
Tumor size					0.006	2.53	(1.25–5.10)	0.01
	≥2.0 cm	49	54.7	47.4				
	<2.0 cm	46	83.5	64.9				
Multiplicity					0.388			
	Solitary	83	69.1	58.6				
	Multiple	12	64.8	34.6				
Macrovascular invasion					0.666			
	Presence	2	50.0	50.0				
	Absence	93	69.1	55.2				
Histology								
Poorly differentiated HCC					0.818			
	Presence	15	62.2	51.8				
	Absence	80	69.9	55.7				
Microvascular invasion					0.077			
	Presence	15	53.3	53.3				
	Absence	80	71.6	55.7				
Liver cirrhosis					0.961			
	Presence	35	72.5	47.3				
	Absence	60	66.4	61.3				

AFP, α-fetoprotein; ALT, alanine aminotransferase; AST, aspartate aminotransferase; BMI, body mass index; CI, confidence interval; DAA, direct-acting antiviral; HCC, hepatocellular carcinoma; HR, hazard ratio; IBT, interferon-based therapy; ICGR 15 min, indocyanine green retention rate at 15 min; PIVKA-II, protein induced by vitamin K absence or antagonist-II; SVR, sustained virological response; UICC, International Union Against Cancer.

**Table 3:** Post-recurrence course in patients with and without alcohol abuse.

	With Alcohol Abuse		Without Alcohol Abuse		
Variables	(n = 8)		(n = 28)		*p* Value
Recurrence sites					1.00
Liver	7	(88)	25	(89)	
Other organs	1	(13)	3	(11)	
Child–Pugh class (A/B) disease at recurrence	7/1		27/1		0.400
Treatments					0.434
Repeat liver resection or ablation therapy	3	(38)	16	(57)	
TACE or other treatments	5	(63)	12	(43)	
Death	2	(25)	9	(32)	1.00
Causes of death					
HCC	2	(25)	8	(29)	
Liver failure	0		1	(4)	
Post-recurrence survival rate (%)					0.79
3 years after surgery	66.7		67.1		
5 years after surgery	66.7		67.1		

TACE, transcatheter arterial chemoembolization.

**Table 4:** Comparison of clinicopathological characteristics in patients with and without alcohol abuse.

	With Alcohol Abuse		Without Alcohol Abuse		
Variables	(n = 10)		(n = 85)		*p* Value
Background					
Age (y.o.)	67	(48–77)	73	(45–88)	0.012
Sex (male/female)		10/0		46/39	0.005
History of IBT	4	(40.0)	24	(28.2)	0.474
BMI (Kg/m^2^)	23.6	(17.2–33.8)	23.3	(17.0–34.8)	0.396
Diabetes mellitus	3	(30.0)	21	(24.7)	0.709
Preoperative liver function tests					
Total bilirubin (mg/dL)	0.8	(0.5–1.5)	0.7	(0.2–2.2)	0.380
Albumin (g/dL)	4.1	(3.3–4.7)	4.2	(3.3–5.0)	0.841
Prothrombin activity (%)	99	(62–114)	91	(59–125)	0.272
ICGR 15 min (%)	14.4	(5.0–26.6)	13	(1.0–37.0)	0.734
Child–Pugh class B	0		1	(1.2)	1.00
Platelets (×10^4^/μL)	13.8	(8.1–21.8)	12.3	(4.1–34.9)	0.658
AST (IU/L)	36	(19–56)	25	(12–52)	0.016
ALT (IU/L)	26	(12–66)	18	(7–52)	0.062
Surgery-related variables					
Segmentectomy or more	4	(40.0)	27	(31.8)	0.724
Minimal invasive surgery	10	(100)	64	(75.3)	0.111
Operation time (min)	334	(147–641)	240	(86–594)	0.011
Bleeding (cc)	130	(15–1800)	50	(0–2340)	0.148
Tumor-related variables					
AFP (ng/mL)	10.4	(1.8–2826)	6.0	(1.0–25452)	0.61
PIVKA-II (U/mL)	48.5	(16–188)	27	(1.0–15600)	0.544
Tumor size (cm)	1.8	(0.7–3.5)	2.0	(0.8–7.5)	0.322
Multiplicity	1	(10.0)	11	(12.9)	1.00
Macrovascular invasion	0		2	(2.4)	1.00
UICC stage 1A/1B/2		5/4/1		40/33/12	0.937
Histology					
Poorly differentiated HCC	1	(10.0)	14	(16.5)	1
Microvascular invasion	2	(20.0)	13	(15.3)	0.656
Liver cirrhosis	3	(30.0)	32	(37.6)	0.741

Note: data are represented as median (ranges) or number (percentages). AFP, α-fetoprotein; ALT, alanine aminotransferase; AST, aspartate aminotransferase; BMI, body mass index; CI, confidence interval; DAA, direct-acting antiviral; HCC, hepatocellular carcinoma; HR, hazard ratio; IBT, interferon-based therapy; ICGR 15 min, indocyanine green retention rate at 15 min; PIVKA-II, protein induced by vitamin K absence or antagonist-II; SVR, sustained virological response; UICC, International Union Against Cancer.

## Data Availability

All data generated or analyzed during this study are included in this article. Further enquiries can be directed to the corresponding author.

## Abbreviations

The following abbreviations are used in this manuscript:

DAA	Direct-acting antiviral
DFS	Disease-free survival
HAI	Histological activity index
HCV	Hepatitis C virus
HR	Hazard ratio
IBT	Interferon-based therapy
OS	Overall survival
SVR	Sustained virological response
UICC	Union for International Cancer Control
